# Molecular modelling studies and in vitro enzymatic assays identified A 4-(nitrobenzyl)guanidine derivative as inhibitor of SARS-CoV-2 Mpro

**DOI:** 10.1038/s41598-024-59292-0

**Published:** 2024-04-14

**Authors:** Kaio Maciel de Santiago-Silva, Priscila Goes Camargo, Larissa Esteves Carvalho Constant, Stephany da Silva Costa, Giovanna Barbosa Frensel, Diego Allonso, Gerson Nakazato, Camilo Henrique da Silva Lima, Marcelle de Lima Ferreira Bispo

**Affiliations:** 1 Laboratório de Síntese de Moléculas Medicinais (LaSMMed), Departamento de Química, Centro de Ciências Exatas, Universidade Estadual de Londrina, Londrina, Brazil; 2 Departamento de Química Orgânica, Instituto de Química, Universidade Federal Do Rio de Janeiro, Rio de Janeiro, Brazil; 3 Departamento de Biotecnologia Farmacêutica, Faculdade de Farmácia, Universidade Federal Do Rio de Janeiro, Rio de Janeiro, RJ 21941-902 Brazil; 4 Departamento de Microbiologia, Centro de Ciências Biológicas, Universidade Estadual de Londrina, Londrina, Brazil

**Keywords:** 3CL protease, Molecular docking, Molecular dynamics, Inhibitors, Coronavirus, COVID-19, Biochemistry, Chemical biology, Computational biology and bioinformatics, Drug discovery, Medical research

## Abstract

Scientists and researchers have been searching for drugs targeting the main protease (Mpro) of SARS-CoV-2, which is crucial for virus replication. This study employed a virtual screening based on molecular docking to identify benzoylguanidines from an in-house chemical library that can inhibit Mpro on the active site and three allosteric sites. Molecular docking was performed on the LaSMMed Chemical Library using 88 benzoylguanidine compounds. Based on their RMSD values and conserved pose, three potential inhibitors (**BZG1**, **BZG2**, and **BZG3**) were selected. These results indicate that **BZG1** and **BZG3** may bind to the active site, while **BZG2** may bind to allosteric sites. Molecular dynamics data suggest that **BZG2** selectively targets allosteric site 3. In vitro tests were performed to measure the proteolytic activity of rMpro. The tests showed that BZG2 has uncompetitive inhibitory activity, with an IC_50_ value of 77 µM. These findings suggest that benzoylguanidines possess potential as Mpro inhibitors and pave the way towards combating SARS-Cov-2 effectively.

## Introduction

The COVID-19 pandemic has affected millions of people worldwide, causing devastating consequences for individuals, families, and communities. The culprit behind this global crisis is the severe acute respiratory syndrome coronavirus-2 (SARS-CoV-2). Since the start of the COVID-19 pandemic over three years ago, there have been 772,011,164 confirmed cases and 6,940,593 deaths globally as of November 2023^1^. Vaccines have been crucial in the fight against COVID-19, but the emergence of new strains like Alpha, Beta, and Omicron pose challenges to the effectiveness of existing vaccines^2,3^. Three drugs have been accepted by the Food and Drug Administration (FDA) and were initially promising as potential pharmacological treatments for COVID-19: Remdesivir^4^, which targets RNA-dependent RNA polymerase (RdRp), has been fully approved for use in adult and pediatric patients 12 years of age and older weighing at least 40 kg requiring hospitalization; Molnupiravir^5^ is currently under emergency use authorization, and Paxlovid^6^ (a combination of Nirmatrelvir and Ritonavir) has been fully approved for treating mild-to-moderate COVID-19 in adults at high risk for severe disease, both targeting main protease (Mpro). However, further research revealed poor outcomes for these drugs. Remdesivir’s clinical efficacy is limited due to its short exposure and half-life influence. Moreover, its distribution may be unequal as specific tissues metabolize it over others preferentially. Clinical studies have demonstrated hepatotoxicity among humans exposed to Remdesivir therapy^7^. Nirmatrelvir has low oral bioavailability as the CYP3A4 enzyme metabolizes it. Therefore, it is administered in a dose of 300 mg with 100 mg of Ritonavir, which inhibits this CYP to ensure sufficient blood concentrations of Nirmatrelvir. However, due to CYP3A4's inhibition, drug interactions may cause severe adverse reactions such as diarrhea, hypertension, and myalgia^8^. Finally, Molnupiravir also presents adverse effects, with diarrhea, nausea, and dizziness being the most common ones^9^, emphasizing the need for new drugs to combat coronavirus.

Nevertheless, drug development is complex and challenging since it requires a significant investment of time and financial support. Computational tools like molecular docking and crystallographic screening are used in virtual screening to identify potential inhibitors from chemical libraries, helping reduce costs and time^10,11^. In this context, the Mpro enzyme of the SARS-CoV-2 virus is a potential target for drug development. Inhibiting this protease can prevent the virus from replicating, making it an essential target for antiviral drugs. Previous research has identified compounds that can effectively block the enzymatic activity of Mpro, thereby inhibiting viral replication^12–16^.

**Figure 1:**
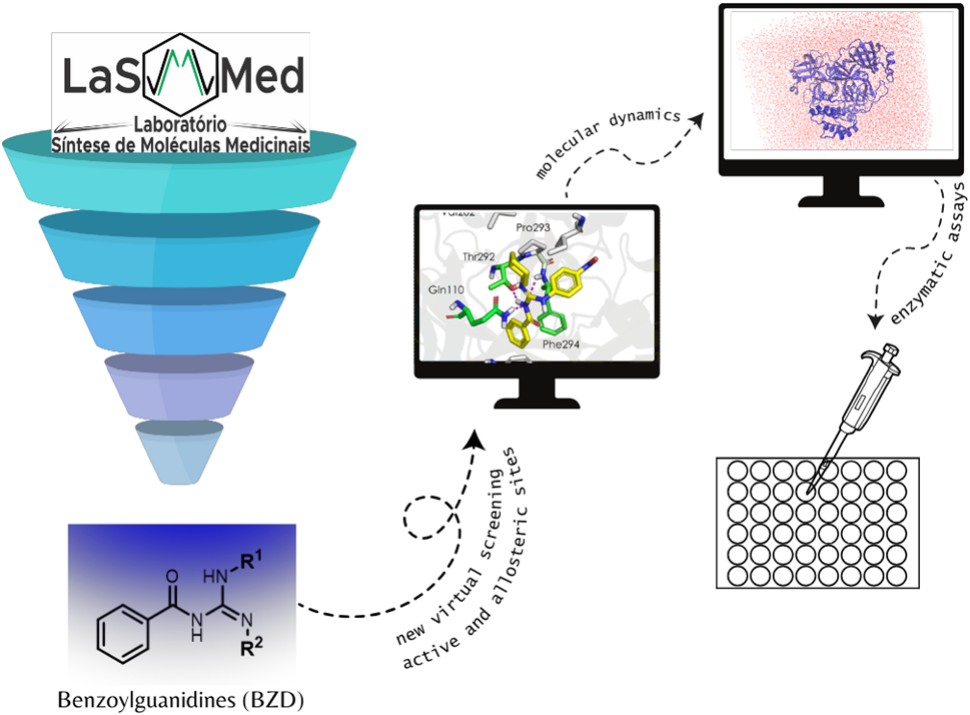
The experimental approach to benzoylguanidine molecular modeling and enzymatic evaluation against Mpro.

Given the potential of Mpro as a molecular target, we explored two different strategies to search for inhibitors in our in-house chemical library (LaSMMed Chemical Library, LMCL) by molecular docking in two Mpro crystallographic structures. In a previous report, our research group conducted a virtual screening of 313 diverse structures from LMCL. Docking and dynamic molecular simulations performed on the active site of Mpro revealed that the top-ranked compounds were benzoylguanidines (BZG)^17^. Therefore, in the current research, we performed a virtual screening of a specific group of 88 BZG derivatives to explore and understand their potential for inhibiting the active or allosteric sites of Mpro. Furthermore, we aimed to identify highly potent compounds that exhibit inhibitory effects in vitro. (Fig. 1).

## Results and discussions

### Molecular modeling studies

We conducted a virtual screening based on molecular docking to investigate the LaSMMed Chemical Library (LMCL) with 88 benzoylguanidines (**BZG**) (Table S1) against two Mpro SARS-CoV-2 structures (6LU7^15^ and 6Y2F^18^) to verify interactions between ligands and target. First, the screening determined if the structures of BZG1-88 could fit into the substrate binding site and interact with the catalytic dyad (His41 and Cys145)^15^. As previously reported, we validated the docking protocol using the redocking method to ensure accuracy^17^.

Molecular docking simulations on Mpro 6LU7 selected the top 20 **BZG** structures based on their higher binding affinities, which ranged between − 8.9 and − 7.8 kcal/mol (Table S2). After analyzing the binding affinities for Mpro 6Y2F, we found that they were lower than the Mpro 6LU7, ranging from − 7.9 to − 7.11 kcal/mol (Table S2). Eleven selected **BZG** showed consensus between the two enzymes(Table S3). Finally, we used Root-mean-square deviation (RMSD) < 2 Å as a metric to rank structures based on the most conserved pose and how closely the lowest binding energy pose resembles the experimental binding mode^19^ to selected three potential inhibitors of SARS-CoV-2 Mpro. The three top-ranked benzoylguanidines we chose were **BZG1**, **BZG2**, and **BZG3** (Table 1).

**Table 1:** Structures of the three ligands selected in the virtual screening, ΔG (kcal/mol), and the RMSD values obtained.

LMed	2D structure	ΔG (kcal/mol) 6Y2F	ΔG (kcal/mol) 6LU7	RMSD (Å)
BZG1		− 7.3	− 8.1	0.764
BZG2		− 7.15	− 7.8	0.936
BZG3		− 7.3	− 8.0	0.588

**Figure 2:**
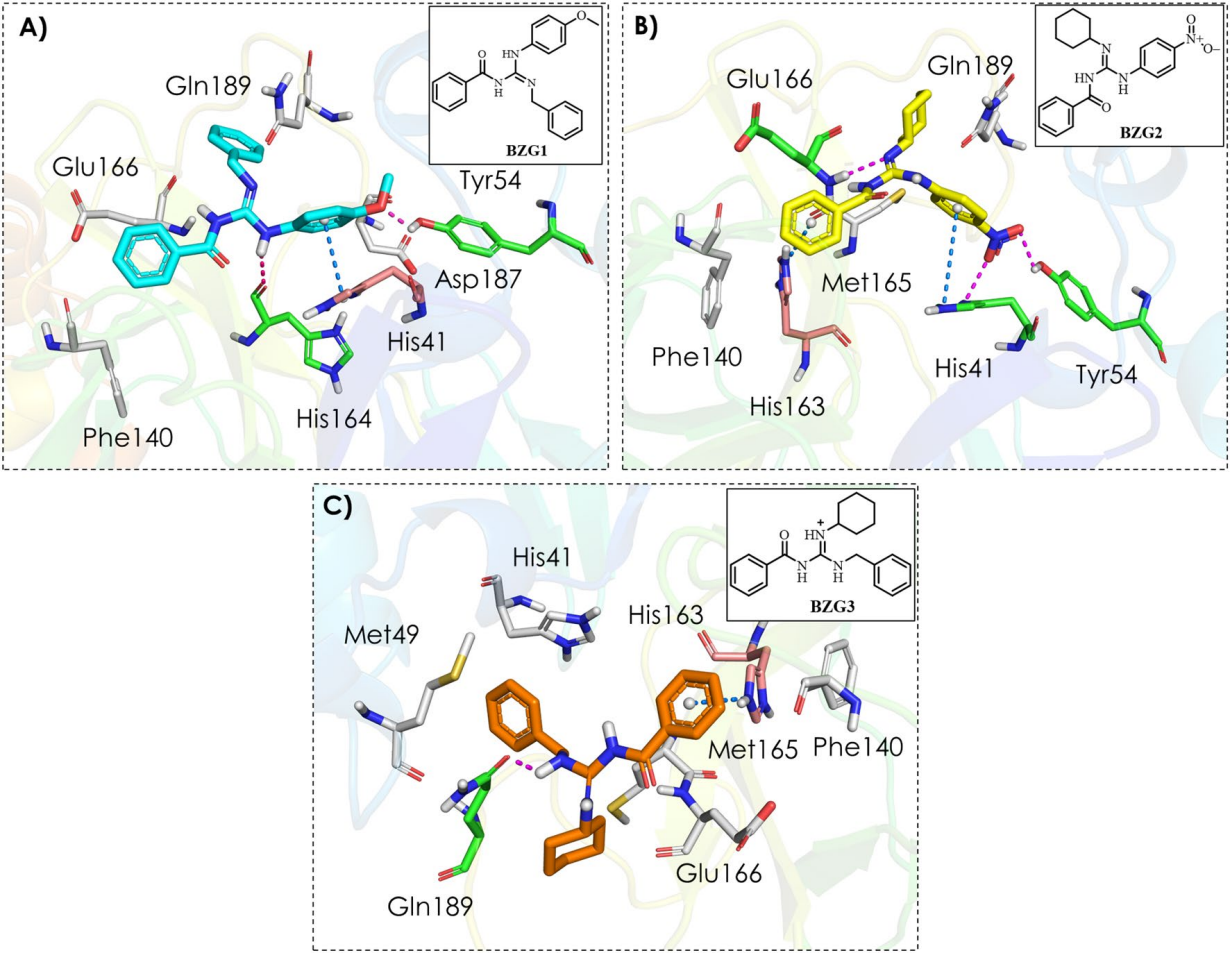
Binding mode and molecular interactions of the three best compounds with the Mpro active site. (**A**) **BZG1** (cyano); (**B**) **BZG2** (yellow); (**C**) **BZG3** (orange). The white amino acid residues represented by sticks carry out hydrophobic interactions with the compounds. Magenta dashed lines represent H-bonds with green amino acid residues, and blue dashed lines represent π-π interactions with salmon amino acid residues.

We observed two hydrogen bonds after analyzing the Mpro-**BZG1** complex (as shown in Fig. 2A). One of these bonds was between the guanidinium group and the oxygen on the main chain of the His164 at the S1 subsite, while the other was between the methoxy group and the side chain of the Tyr54 at the S2 subsite. In addition to these hydrogen bond interactions, we also observed π-π stacking between the ligand and the imidazole ring of the catalytic histidine (His41), as well as hydrophobic interactions with residues Phe140 at the S1 subsite, Asp187 at the S2 subsite, Glu166, and Gln189 at the S4 subsite.

The **Mpro-BZG2** protein–ligand complex (Fig. 2B) has an H-bond between the guanidinium group and Glu166's amide nitrogen at the S4 subsite. This interaction is significant because Glu166 is responsible for Mpro’s dimerization, essential for the enzyme's catalytic activity^20^. Additionally, there are two other hydrogen bonds between the nitro group and the side chains of Tyr54 and catalytic histidine (His41) residues at the S2 subsite, and two π-cation interactions between the ligand and histidines (His163 and His41) at the S1 and S2 subsites, respectively. Furthermore, hydrophobic interactions occur with Ph140 at the S1 subsite, Met165 at the S2 subsite, and Gln189 at the S1 subsite.

The **Mpro-BZG3** complex (Fig. 2C) showed only one H-bond interaction between the guanidinium group and the oxygen of the primary chain of residue Gln189 at the S4 subsite. Along with this hydrogen bond, there was also a π-cation interaction with His163 at the S1 subsite, as well as hydrophobic interactions with Phe140 at the S1 subsite, Met49, Met165, and catalytic histidine (His41) at the S2 subsite, and Glu166 at the S4 subsite.

We conducted a molecular dynamics simulation (MDS) with **BZG1**, **BZG2**, and **BZG3** to assess the behavior of these compounds in the aqueous system for 200 ns using GROMACS software^21^ applying Charmm36 force field^22^.

**Figure 3:**
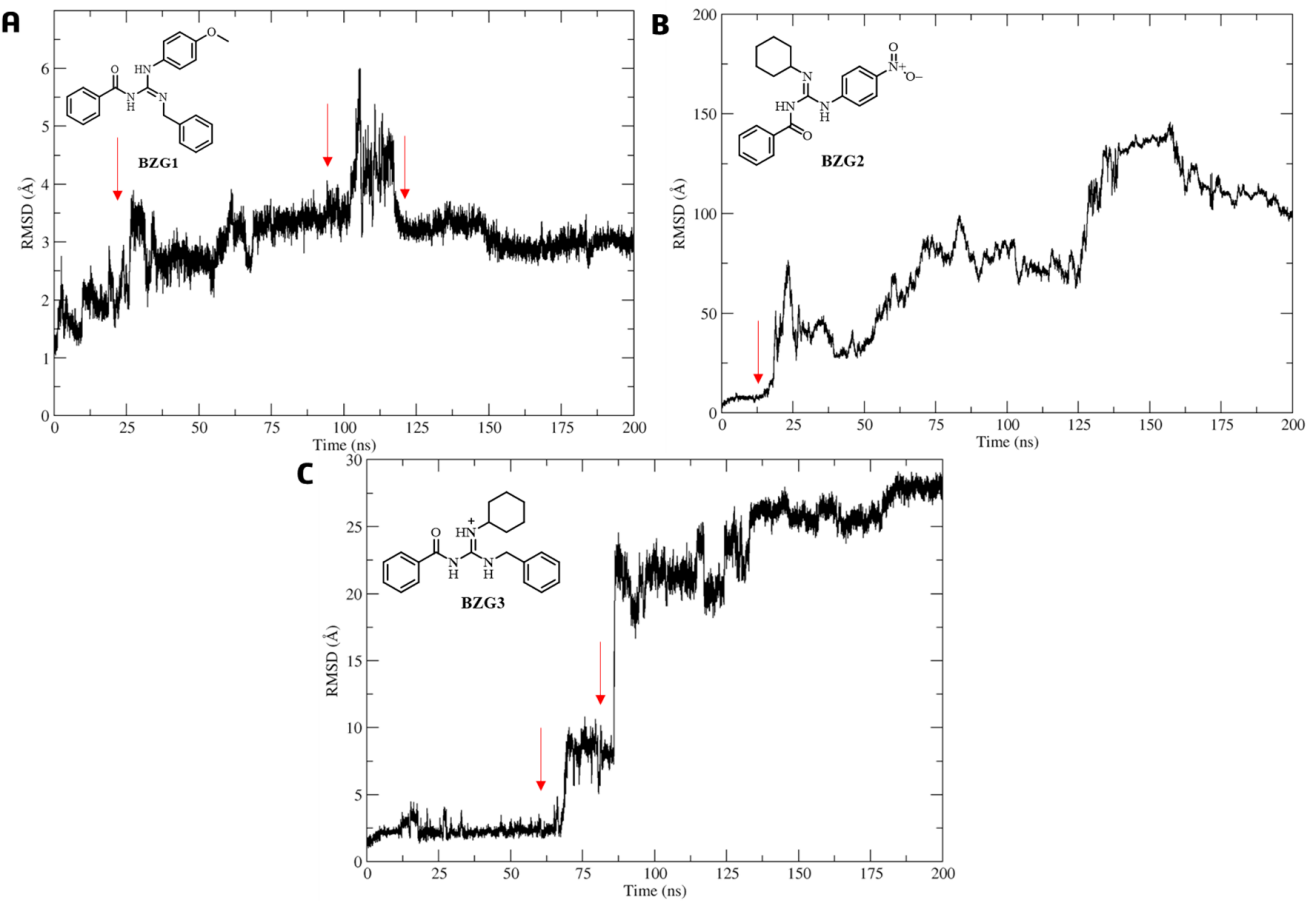
RMSD analysis of the **BZG1** (**A**), **BZG2** (**B**), and **BZG3** (**C**) relative to the Mpro-Cα atoms, the red arrow highlights the movement in the active site.

During the first nanoseconds of MD simulation, ligand **BZG1** remained stable in the active site as per the analysis of RMSD concerning the Cα atoms in the Mpro complex. Although there was slight movement compared to its initial molecular docking pose at 25, 100, and 125 ns, the RMSD value was only 3.03 Å with a low standard deviation (SD) of 0.63 Å, as shown in Fig. 3A. On the other hand, **BZG2** tended to leave the site altogether from 12 ns of MDS with an RMSD value of 77.96 ± 37.82 Å and a high standard deviation (SD) (Fig. 3B). In comparison, **BZG3** remained stable during the first 70 ns of MDS and also exhibited a movement between 70 and 85 ns, then leaving the active site with an RMSD value of 15.42 ± 10.67 Å and a high SD (Fig. 3C).

**Figure 4:**
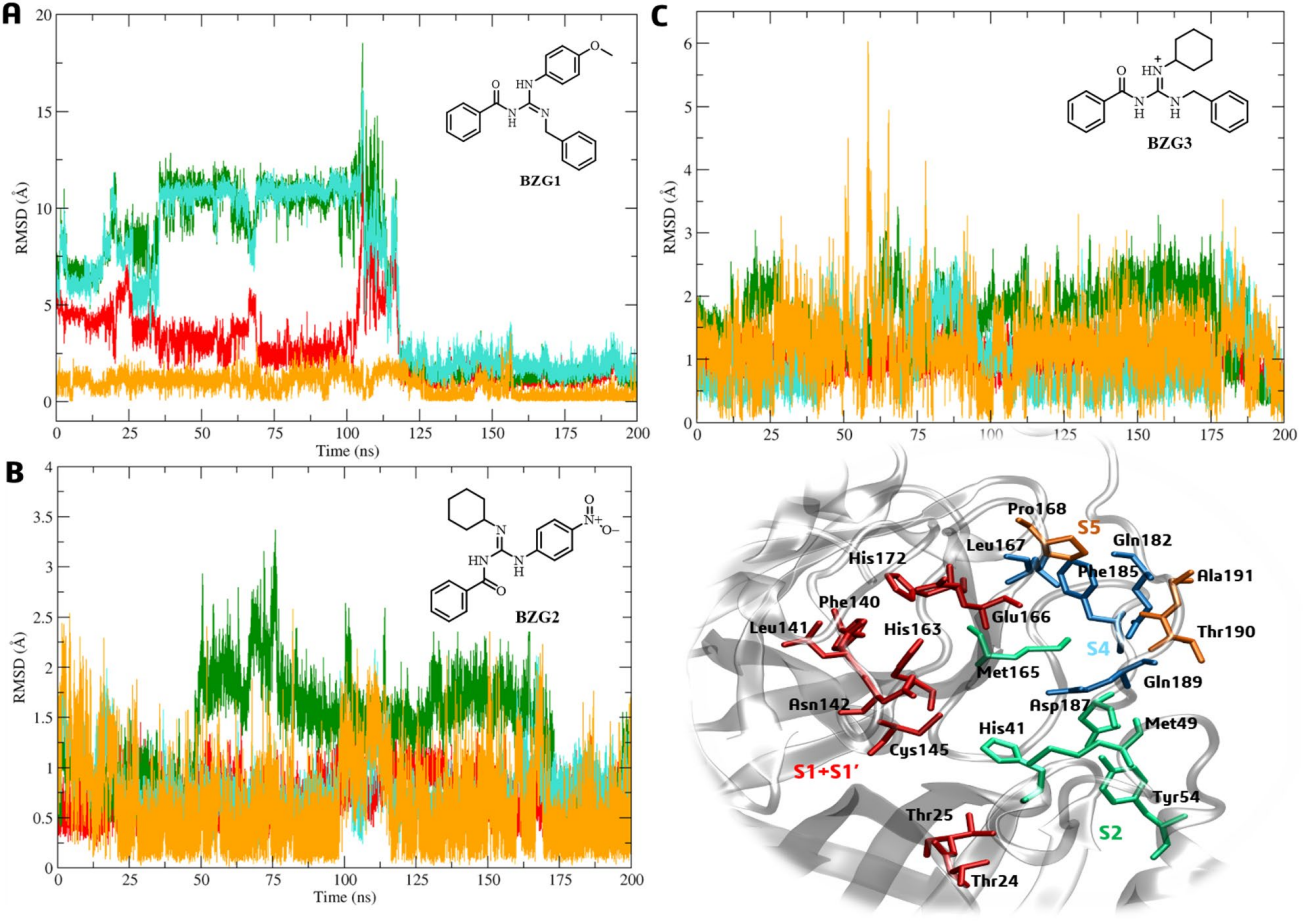
Cα-RMSD analysis to Mpro subsites S1 + S1’ (red), S2 (green), S4 (light blue), and S5 (orange) relative to binding with **BZG1** (**A**), **BZG2** (**B**), and **BZG3** (**C**).

We analyzed the RMSD profiles of the Cα atoms for subsites S1 + S1′, S2, S4, and S5. The Cα atoms for the **BZG1-Mpro** complex remained stable with minimal variation in SD values (< 1.0 Å)^23^ for subsite S5 and RMSD = 0.89 ± 0.53 Å. However, in the first 100 ns of the MD simulation, it was observed that S2 and S4 had high mean and SD values of 6.25 ± 4.23 and 6.25 ± 4.00 Å, respectively (Fig. 4A). This finding aligned with the RMSD profile for **BZG1**, as discussed above. The ligand binding in the active site affected the movement in S2 and S4 subsites. A movement trend was observed in the Cα atoms from the S1 + S1′ subsites, presenting an RMSD of 2.77 ± 1.70 (Fig. 4A).

We observed that the formation of the **BZG2-Mpro** complex is unstable, as shown by the low RMSD and SD values for all subsites. The Cα atoms had values of 0.71 ± 0.20 Å (S1 + S1′), 1.34 ± 0.52 Å (S2), 0.77 ± 0.29 Å (S4), and 0.62 ± 0.43 Å (S5). The ligand output in the first ns of simulation (Fig. 4B) reflects this instability. During the simulation, the **BZG3-Mpro** complex displayed RMSD values of 1.00 ± 0.20 Å (S1 + S1′), 1.64 ± 0.51 Å (S2), 1.05 ± 0.60 Å (S4), and 1.16 ± 0.55 Å (S5), with the S2 subsite showing the most significant variations, even with low mean values (Fig. 4C).

**Figure 5:**
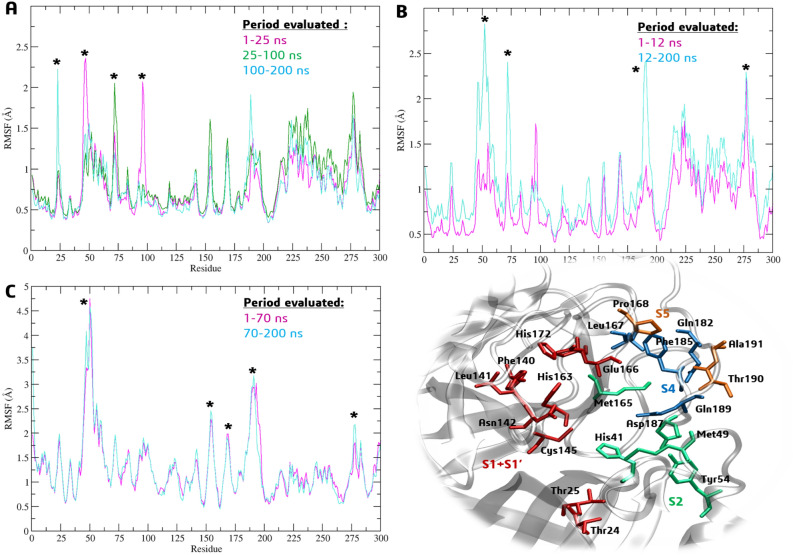
Cα-RMSF analysis for Mpro complexes at different times relative to binding with **BZG1** (**A**), **BZG2** (**B**), and **BZG3** (**C**). The * symbol indicates fluctuations ≥ 2.00 Å.

We compared the root-mean-square-fluctuation (RMSF) of Cα atoms considering the movement of ligands during the 200 ns of simulation; we evaluated the difference in time intervals: 1–25, 25–100 ns and 100–200 ns to **BZG1**; 1–12 and 12–200 to **BZG2**; 1–70 and 70–200 ns to **BZG3** (Fig. 5A–C). We observed that ligand **BZG1** increased the mobility of Met49, Leu75, and Pro96 at the S2 subsite during the first 100 ns of MDS, with RMSF values of 2.4 Å, 2.0 Å, and 2.0 Å, respectively. Following this period, only fluctuations in Thr25 at the S1 subsite were observed with an RMSF value of 2.2 Å (Fig. 5A). This observation aligns with the RMSD analysis, which indicates that the presence of the ligand has the most significant impact on the S1 and S2 subsites.

During the simulation, the **BZG2** compound was initially linked to the center of the active site, which is close to the His41-Cys145 catalytic diade. After 12 ns of MDS, ligand **BZG2** induced RMSF fluctuations of at least 2.0 Å in residues Tyr54 and Thr190 from S2 and S5 subsites, respectively. Also, residues Ser46, Asn51, Asn72, and Met276 outside the active site also experienced these fluctuations, as shown in Fig. 5B. Interestingly, both Thr25 and Tyr190 form the active site's border, and the fluctuations caused by **BZG2** suggest this ligand lacks the affinity to remain connected to the center of the site.

We have analyzed the RMSF for **BZG3** before (1–70 ns) and after (70–200 ns) the movement on the active site as shown in Fig. 5C. The ligand has induced higher mobility in specific amino acids during the analyzed periods. These include Tyr154 (RMSF = 2.46 Å), Asn277(RMSF = 2.17 Å) and residues from two subsites: Met49 (S2, RMSF = 4.75 Å), Pro168 (S5, RMSF = 1.98 Å) and Ala191 (S5, RMSF = 3.17 Å), with the last one belonging to active site^14^.

**Figure 6:**
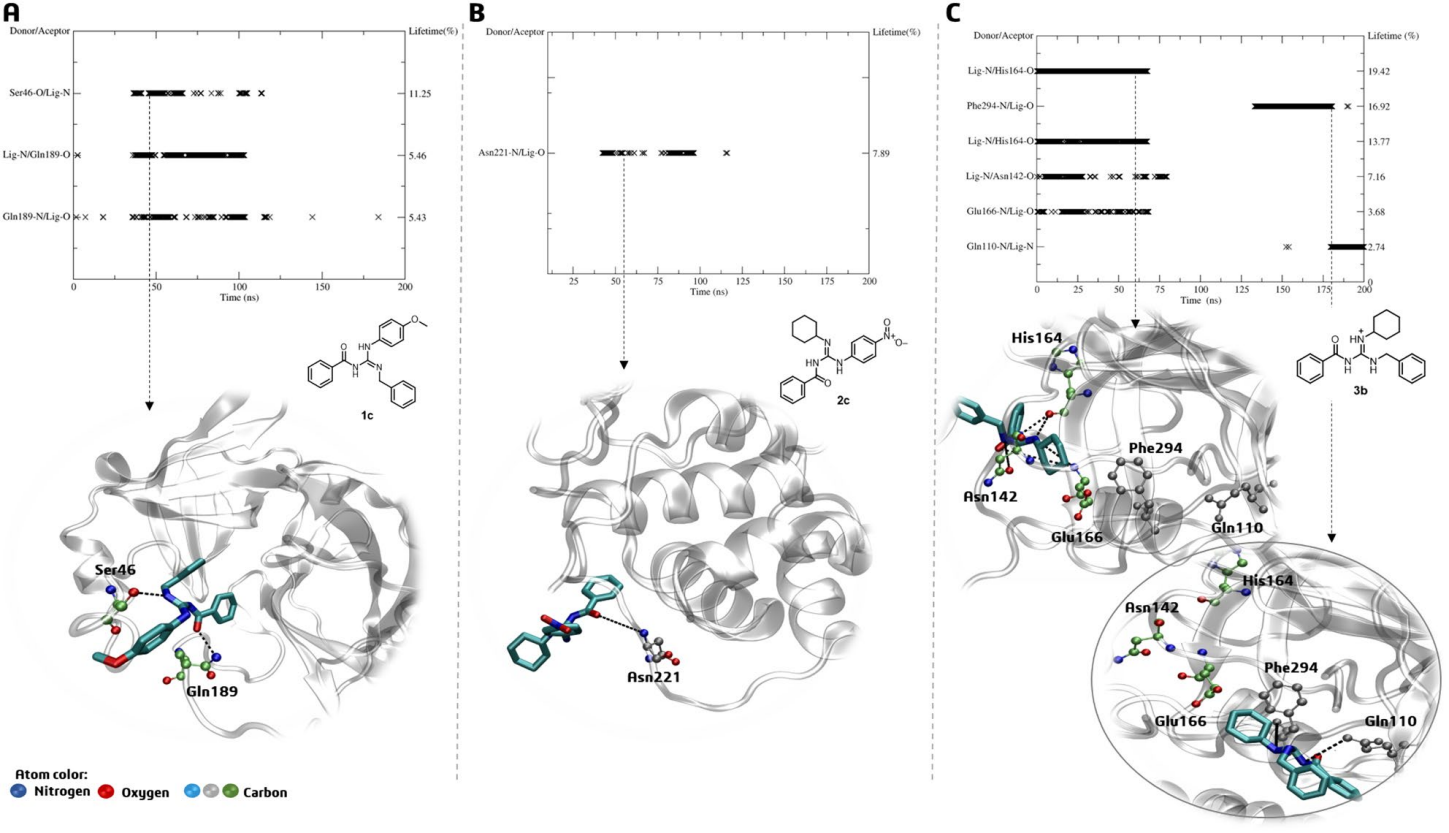
H-bond lifetime (%) and representative interactions of (**A**) **BZG1**, (**B**) **BZG2,** and (**C**) **BZG3-Mpro** complexes in the time indicated by the dashed arrow.

**BZG1**, **BZG2**, and **BZG3-Mpro** complexes were analyzed to determine their intermolecular interactions through H-bonding. The analysis revealed that compound BZG1 could establish an H-bond with the amino acid Gln189 at the S1 subsite and residue Ser46 during 50–125 ns of MDS. This interaction demonstrated a short lifetime of about 5 to 11%, as shown in Fig. 6A. It is worth noting that the interaction mentioned earlier impacted the stability of the amino acids in the S2 subsite, as indicated by the RMSF analysis. However, derivative **BZG2** only formed a hydrogen bond with Asn221, which is far from the active site. Additionally, the bond had a lifetime of only 7.8% between 50 and 125 ns of MDS, as shown in Fig. 6B. This finding aligns with the RMSD analysis, indicating that this derivative possibly lacked an affinity for the active site. **BZG3** had the most consistent interactions (3–19%) with Glu166, Asn142, and His164 residues at the S1 subsite during the first 75 ns of MDS, resulting in fluctuations of residues belonging to subsites S2 and S5, as indicated by RMSF values. Later, **BZG3** interacted with Phe294 and Gln110, further away from the initial residues (Fig. 6C).

Based on the persistence of H-bonds and the analysis of RMSD and RMSF of ligands **BZG1** and **BZG3**, it is more apparent that these compounds have an affinity for Mpro’s active site. It is because they presented the best ΔG_bind_ values during the period in which they remained stable and attached to the enzyme. Ligand **BZG1** had a ΔGbind value of − 39.9 kcal/mol, while ligand **BZG3** had a ΔG_bind_ value of − 18.8 kcal/mol. On the other hand, ligand **BZG2** showed an unfavorable ΔG_bind_ value of 3.46 kcal/mol, according to Table 2.

**Table 2:** The binding free energy (ΔG_bind_) terms of the ligand-Mpro complexes were calculated for **BZG1**, **BZG2**, and **BZG3** (mean ± standard deviation; kcal/mol).

Compound	Time (ns)	ΔE_vdw_	ΔE_elect_	ΔE_solv_	ΔE_sasa_	ΔG_bind_
BZG1	1–25	* − *26.9 ± 4.20	* − *3.43 ± 1.30	5.45 ± 0.63	* − *3.53 ± 0.26	* − *28.4 ± 4.66
	25–100	* − *31.6 ± 2.35	* − *1.09 ± 1.16	6.48 ± 1.23	* − *3.69 ± 0.23	* − *29.9 ± 2.75
	100–200	* − *36.9 ± 2.16	* − *4.46 ± 1.07	5.52 ± 0.40	* − *4.02 ± 0.20	* − *39.9 ± 2.20
BZG2	1–12	0	* − *0.07 ± 0.02	0.67 ± 4.22	0.09 ± 0.38	0.69 ± 4.34
	12–200	0	* − *0.02 ± 0.01	3.47 ± 3.87	0.02 ± 0.53	3.46 ± 3.91
BZG3	1–70	* − *1.01 ± 0.25	0.70 ± 0.32	2.25 ± 1.11	* − *0.35 ± 0.11	1.59 ± 0.96
	70–200	* − *26.4 ± 1.86	* − *0.20 ± 0.84	11.2 ± 1.76	* − *3.39 ± 031	* − *18.8 ± 1.44

ΔEvdW = Van der Waals, ΔEelect = electrostatic, ΔEsolv = solvation, ΔEsasa = solvent accessible surface area.

According to our findings, the **BZG1** and **BZG3** exhibit a strong affinity towards the subsites of the Mpro enzyme. This affinity is due to the hydrophobic nature of the residues that compose the subsites, particularly Phe140, Leu141 (forming subsite S1), and Met49, Tyr54, and Met165 (forming subsite S2). When diverse hydrophobic substituents, like phenyl, benzyl, p-methoxyphenyl, and cyclohexyl groups, are present in the ligand structure, the interaction with these pockets becomes more robust. It is clear from the Van der Waals energies, which range from − 26 to − 36 kcal/mol for both compounds that they have a stronger binding affinity to the Mpro active site than nitro-substituted **BZG2**, which does not show this contribution. Additionally, **BZG2** does not provide favorable energy values regarding electrostatics, solvation, or solvent-accessible surface area, further emphasizing its poor affinity for the Mpro active site (Table 2). To investigate the possibility of **BZG2** exhibiting uncompetitive inhibition behavior, we evaluated its affinity in three possible allosteric sites reported by DasGupta et al^24^. The allosteric site 1 (**A1**) comprises hydrophobic residues such as Gln107, Pro108, Gly109, Ile200, Thr201, Val202, Asn203, Ile249, Pro252, Leu253, Phe294, Asp295 and Val297. Allosteric site 2 (**A2**) is a compact, formed by amino acid Phe3, Ile213, Asn214, Gln299, Cys300, Ser301, Tyr118, Phe140, and Leu141. Finally, allosteric site 3 (A3) is composed of residues Leu271, Leu272, Gly275, Met276, and Asn277^24^.

**Figure 7:**
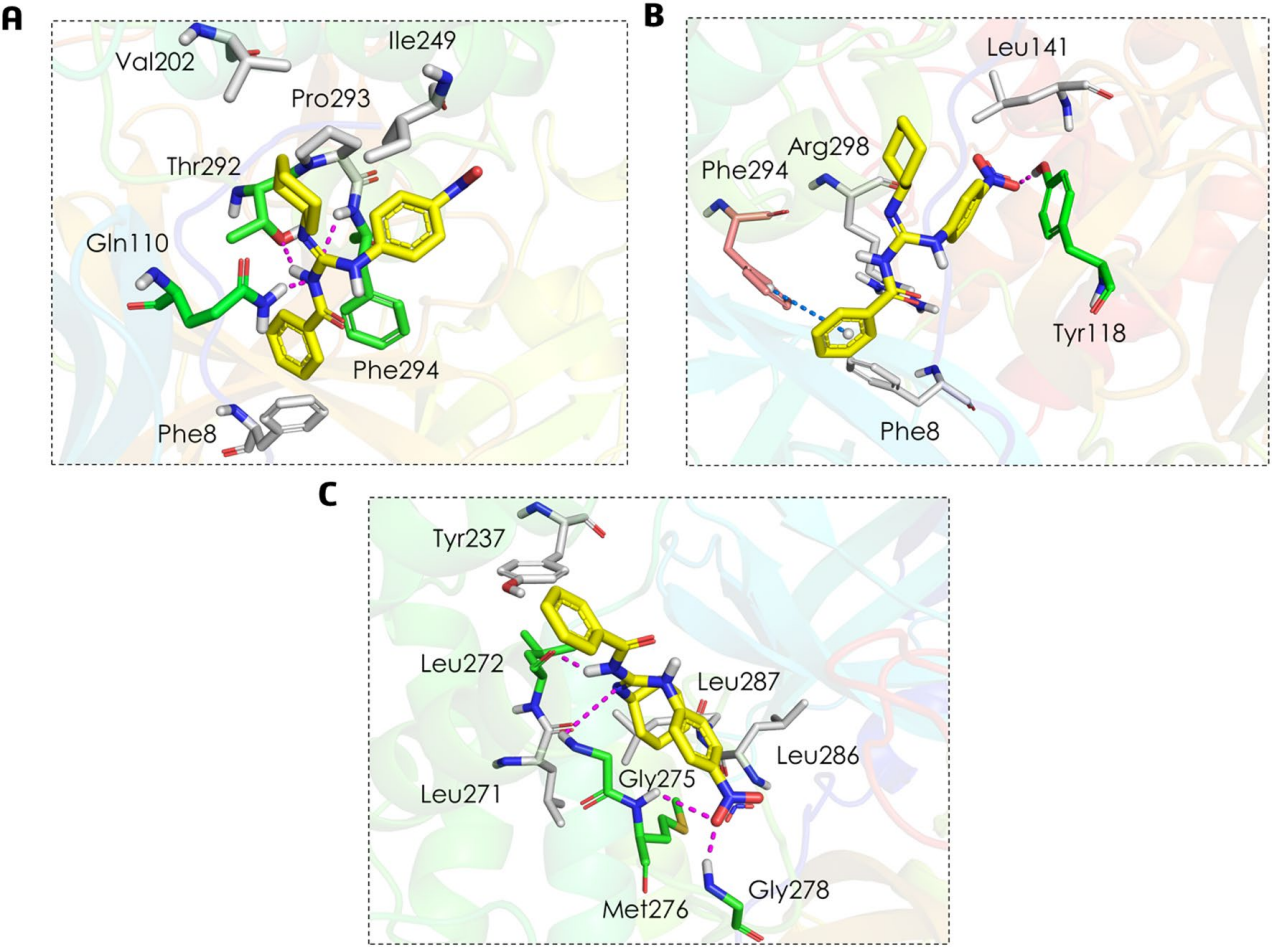
Binding mode and molecular interactions of **BZG2** ligand within the three allosteric sites: (**A**) A1, (**B**) A2, and (**C**) A3. The white amino acid residues represented by sticks carry out hydrophobic interactions with the compounds. Magenta dashed lines represent H-bonds with green amino acid residues, and blue dashed lines represent π interactions with salmon amino acid residues.

After performing molecular docking and analyzing the complex between ligand **BZG2** at the A1 site (as shown in Fig. 7A), we observed three hydrogen bonds involving the guanidinium group with the main chains of residues Gln110 and Phe294, as well as with the side chain of residue Thr292. Additionally, we observed hydrophobic interactions with residues Phe8, Val202, IIe249, and Pro293.

At site A2, the ligand **BZG2** (Fig. 7B) forms a single hydrogen bond with the Tyr118 residue. It also interacts through a π-π T-shaped interaction with the aromatic ring of residue Phe294 and through hydrophobic interactions with residues Phe8, Leu141, and Agr298. On the other hand, based on Fig. 7C, it seems that **BZG2** has a stronger affinity to the A3 site. It is due to the formation of four hydrogen bonds, two between the guanidinium group and the main chain of residue Leu272 and the side chain of Gly275. At the same time, the other two occur between the nitro group and the main chain of Met276 and Gly278 residues. Additionally, **BZG2** exhibits hydrophobic interactions with Tyr237, Leu271, Leu286, and Leu287 residues.

**Figure 8:**
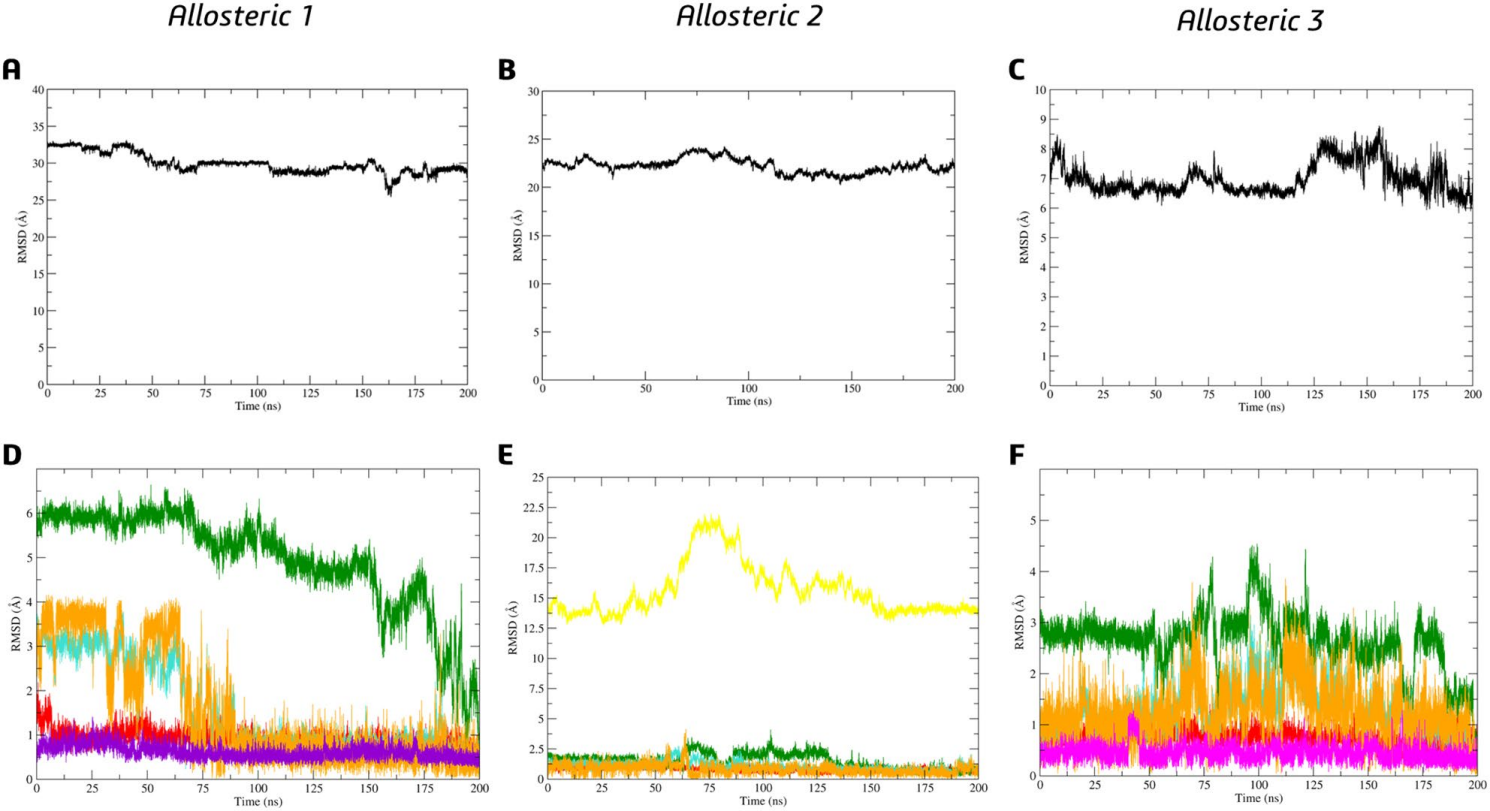
Ligand **BZG2** RMSD analysis in the allosteric sites (**A**) A1; (**B**) A2 and (**C**) A3. Cα-RMSD analysis to Mpro sites S1 + S1’ (red), S2 (green), S4 (blue), S5 (orange), A1 (purple), A2 (yellow), and A3 (pink) relative to binding of (**D**) **BZG2-A1**; (**E**) **BZG2-A2**; (**F**) **BZG2-A3**.

In order to investigate the possibility of selectivity, we conducted molecular dynamic simulations on **BZG2** complexes at the three allosteric sites. Based on the analysis of RMSD concerning **BZG2** on allosteric complexes, we discovered that this particular ligand displayed a lower value of RMSD and SD to A3, approximately 6.99 ± 0.52 Å (as shown in Fig. 8C). However, for allosteric sites A1 and A2, the values were relatively higher at 29.95 ± 1.38 Å (as shown in Fig. 8A) and 22.24 ± 0.76 Å (as shown in Fig. 8B), respectively, when compared to their initial docking pose (as depicted in Fig. 7B).

We noticed that the RMSD values of the Cα atoms in the allosteric sites were affected by the presence of the ligand **BZG2** in the A1 cavity, even after it had left. Specifically, we found that the RMSD values for subsites S2 remained high (4.93 ± 1.17 Å) throughout the entire MDS period, indicating a significant impact of the ligand on this subsite. During the first 75 ns, the presence of the ligand affected the S4 and S5 subsites with RMSD values of 1.53 ± 0.99 Å and 1.55 ± 1.28 Å, respectively, as illustrated in Fig. 8D. However, there were no noticeable changes in the RMSD values for the subsites of A2. On the other hand, significant differences were observed for the Cα atoms of A2, particularly between 75 and 125 ns, with a value of 15.66 ± 2.16 Å, despite the ligand leaving the cavity of this allosteric site, as shown in Fig. 8E. About the analysis of A3, the most significant RMSD variations were observed for subsites S2 (2.63 ± 0.57 Å), followed by subsites S4 (1.31 ± 0.49 Å) and S5 (1.23 ± 0.51 Å). However, there was no significant variation for A3 (0.45 ± 0.16 Å), as shown in Fig. 8F.

We also performed an RMSD analysis for Cα atoms of the Mpro in its apo form (Fig. S1), which demonstrated the values for S1 + S1' = 0.83 ± 0.21 Å, S2 = 1.72 ± 0.41 Å, S4 = 1.77 ± 0.44 Å, S5 = 0.79 ± 0.24 Å, A1 = 0.62 ± 0.17 Å, A2 = 1.43 ± 0.34 Å, A3 = 0.92 ± 0.62 Å. Our analysis of the complexes shows that **BZG2** might have a high affinity for allosteric site A3 since it has been proven stable. Furthermore, the interaction of **BZG2** with the surrounding area of A2 can potentially have a negative impact on subsites S2, S4, and S5, which is unfavorable for catalytic activity in both cases.

**Figure 9:**
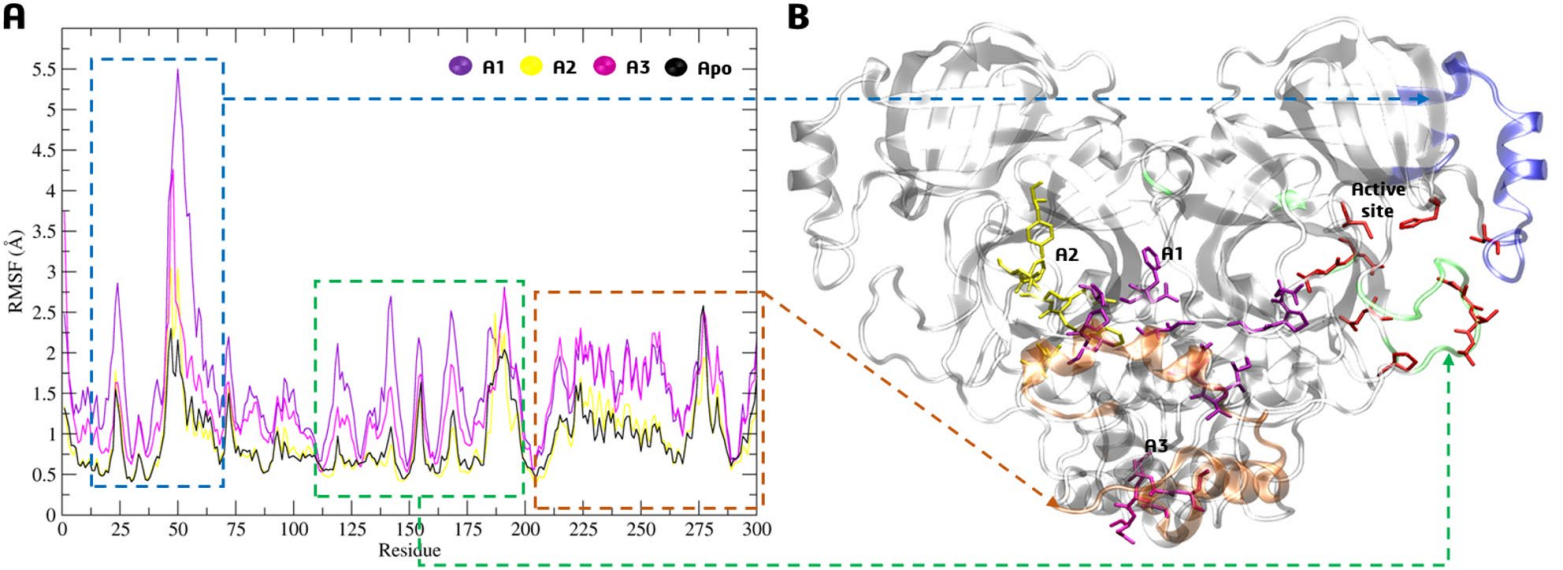
(**A**) Cα-RMSF analysis for complexes between **BZG2** and allosteric sites A1, A2, A3, and the apoenzyme form. (**B**) 3D-Mpro structure highlighting the sites and mainly residues fluctuations.

We analyzed the RMSF of Cα atoms for three allosteric sites compared to the enzyme in its apo form. The fluctuations were particularly noticeable when the ligand was complexed at sites A1 and A3, with variations of up to 6 Å (Fig. 9A) for the amino acids present in the active site (in red, Fig. 9B) and its surrounding areas (in green and blue, Fig. 9B), even though it did not remain connected. Furthermore, fluctuations > 2 Å were also observed for residues near the A1 and A3 region (in orange, Fig. 9B) for these two allosteric sites. However, RMSF for A2 showed no significant variations compared to the apoenzyme form (Fig. 9A).

**Figure 10:**
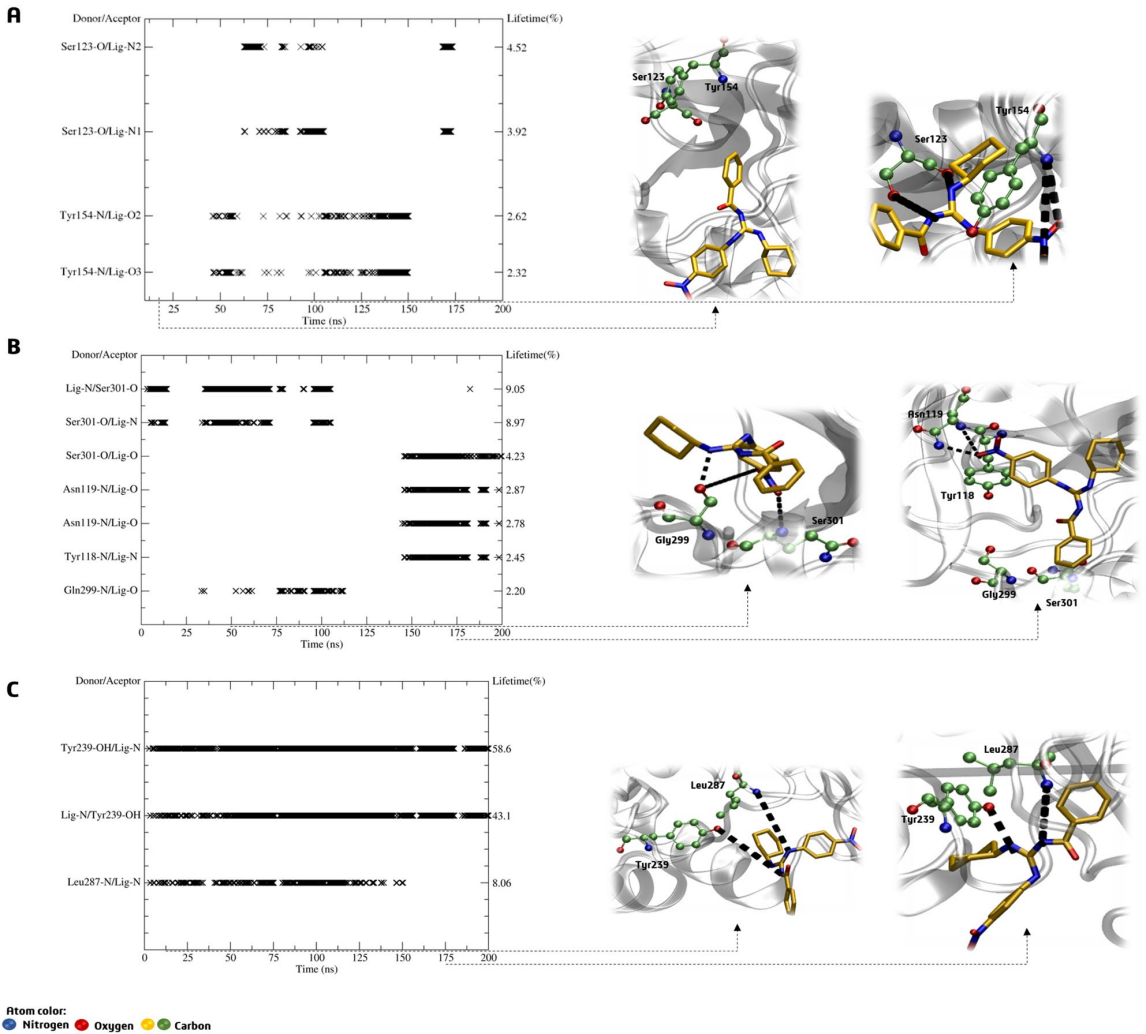
H-bond lifetime (%) and representative interactions of (**A**) **BZG2-A1**, (**B**) **BZG2-A2** and (**C**) **BZG2-A3** allosteric sites complexes in the time indicated by the dashed arrow.

Upon further analysis of the hydrogen bonds, it has become apparent that compound **BZG2** lacks affinity for the A1 allosteric site due to it only interacting briefly with Ser123 and Tyr154 between 50 and 150 ns, with a short lifetime of 2 to 4%, as shown in Fig. 10A. During the first 100 ns of the MDS period, there was a brief lifetime of 2% between the ligand and Ser301 and Gln299 for **BZG2-A2**. In the final 50 ns, interactions occurred with Asn119 and Tyr118 (Fig. 10B). On the other hand, **BZG2-A3** exhibited stronger hydrogen bonding interactions with a high lifetime of up to 58%. This interaction was observed throughout the MDS period with Tyr239 and Leu287 (Fig. 10C). The guanidinium group acted as a bidentate binder with the two amino acids.

The RMSD values of the Cα atoms indicate that the **BZG2** may have a stronger affinity for the A3 allosteric site than the other two sites. The results showed stable values with fluctuations above 6 Å for the A3 site, which suggests higher selectivity. The dynamics studies revealed that **BZG2** shows an uncompetitive behavior, indicating a stronger affinity for the A3 site than the active site.

When comparing the results of ΔG_bind_ for the active site and three allosteric sites, it was found that the active site had an unfavorable positive value of 3.46 kcal/mol (as shown in Table 2). On the other hand, all three allosteric sites exhibited a negative ΔG_bind_ (as indicated in Table 3). It was observed that A1 had the lowest ΔG_bind_ value of − 28.9 kcal/mol, while A2 had a ΔG_bind_ value of -33.2 kcal/mol. It is consistent with the hydrogen bonding analysis that occurred briefly between the target and sites A1 and A2, as shown in Table 3. The **BZG2-A3** complex has a high ΔG_bind_ value of − 34.0 kcal/mol, confirming **BZG2**’s selectivity for the allosteric A3 (Table 3), which also agrees with all other findings.

**Table 3:** The binding free energy (ΔG_bind_) terms of the ligand-allosteric sites complexes were calculated (mean ± standard deviation; kcal/mol).

Complex	Time (ns)	ΔE_vdw_	ΔE_elect_	ΔE_solv_	ΔE_sasa_	ΔG_bind_
BZG2-A1	1–200	* − *30.6 ± 1.39	* − *1.37 ± 0.59	6.81 ± 0.76	* − *3.80 ± 0.13	* − *28.9 ± 1.97
BZG2-A2	1–200	* − *32.7 ± 2.74	* − *3.04 ± 1.04	6.43 ± 0.89	* − *3.92 ± 0.26	* − *33.2 ± 2.91
BZG2-A3	1–200	* − *33.7 ± 2.48	* − *3.50 ± 0.56	7.11 ± 0.74	* − *3.88 ± 0.19	* − *34.0 ± 2.47

ΔEvdW = Van der Waals, ΔEelect = electrostatic, ΔEsolv = solvation, ΔEsasa = solvent accessible surface area.

**BZG2** interacted favorably with serine, leucine, and tyrosine residues through hydrophobic interactions by its aromatic groups. This resulted in Van der Waals energy values of approximately − 30 kcal/mol, which were favorable. In contrast, the active site did not contribute to these interactions. However, the energy required to remove the water molecules from the binding site, around 7 kcal/mol, was not higher than the Van der Waals energy. Moreover, the nitro group present in **BZG2** contributed partially to the electrostatic energy (Table 3).

### Inhibition of rMpro activity by BZG1, BZG2 and BZG3

To experimentally verify the hypotheses formulated through molecular modeling studies, we conducted SARS-CoV-2 Mpro inhibition assays with **BZG1**, **BZG2**, and **BZG3** derivatives previously selected by molecular docking studies. We assessed the inhibition of Mpro protease activity using a FRET-based fluorescent assay with the substrate DABCYL-AVLQSGFRK-EDANS, which refers to the nsp4-nsp5 cleavage site.

**Figure 11:**
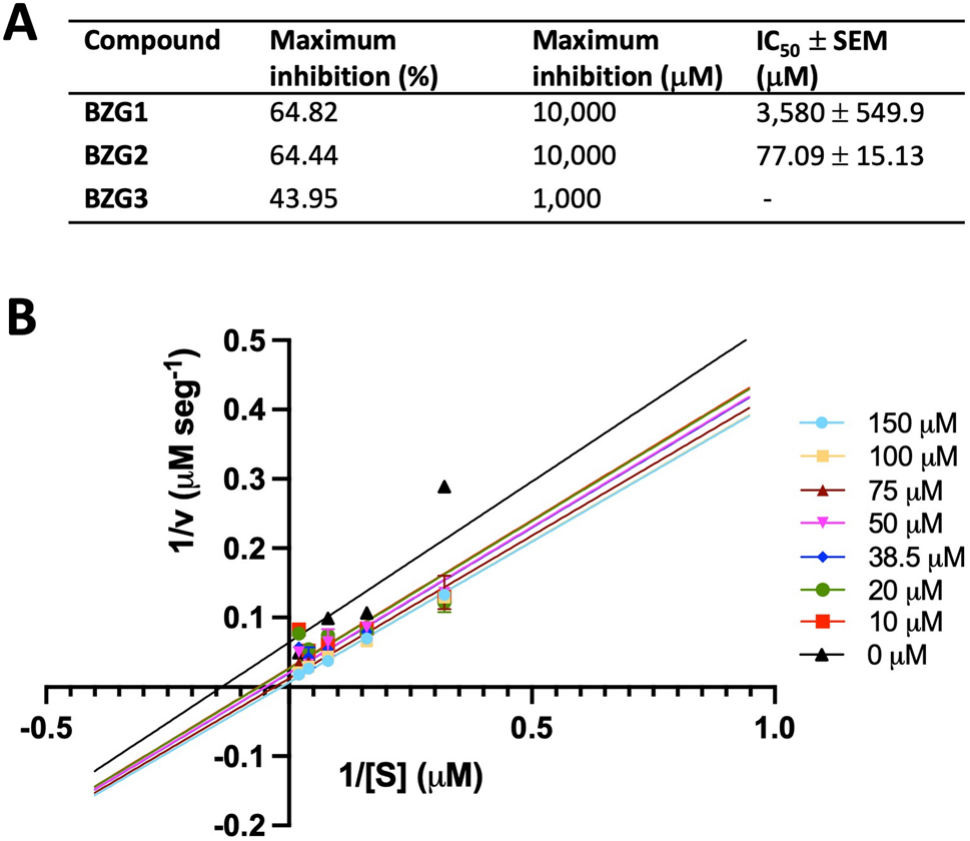
Mpro inhibition assay. (**A**) BZG1, BZG2, and BZG3 inhibition activity was measured against the control condition (vehicle). The percentage of inhibition was calculated assuming that the Mpro cleavage rate at the control condition was 100%. The compound contraction in which maximum inhibition was achieved was set as the concentration of maximum inhibition. IC_50_ ± SEM values were obtained based on the reduction of Mpro proteolytic activity. (**B**) Lineweaver–Burk plot of Mpro inhibition by **BZG2**. All experiments were performed as four independent experiments.

Compounds **BZG1** and **BZG2** exhibited the highest inhibitory capacity against Mpro, with approximately 64% inhibition at the highest tested dose, as shown in Fig. 11A. However, **BZG2** showed a better IC_50_ value than **BZG1**, with 77.09 μM against 3,580 μM, respectively. It is worth noting that **BZG3** had a poor inhibition capacity (< 50%), which made it difficult to calculate the IC_50_ value (Fig. 11A). These results indicate that incorporating a 4-nitrophenyl group on the guanidine nitrogen enhances the inhibitory activity of **BZG2** against Mpro. The change to benzyl in compound BZG3 leads to a significant loss of inhibition capacity. The **BZG1** compound’s inhibitory activity was reduced by tenfold due to the di-substitution of nitrogen atoms by benzyl and 4-methoxyphenyl groups compared to **BZG2**. During molecular docking predictions, we observed significant hydrogen bond interactions between the catalytic residues Cys145 and His41 and the oxygen atoms from the nitro group of **BZG2**’s 4-nitrophenyl moiety. Further, molecular dynamics studies revealed that **BZG2** has a stronger interaction with allosteric site 3 from Mpro than the active site. The guanidinium group in **BZG2** binds to Tyr239 and Leu287 amino acids as a bidentate binder, which could be the reason for its better inhibition potential. In addition, replacing the benzyl ring in **BZG3** and the 4-methoxyphenyl and benzyl groups in **BZG1** resulted in the loss of these interactions.

We conducted an inhibition kinetics study with **BZG2** to understand how it inhibits Mpro activity. The Lineweaver–Burk plot revealed that **BZG2** inhibits Mpro activity through an uncompetitive mechanism (Fig. 11B), which supports our previous molecular in silico results.

### In silico drug-likeness, pharmacokinetic, and toxicity parameters (ADME-Tox) predictions

To predict the oral bioavailability, permeability profile, and drug-likeness of the selected compounds (**BZG1**, **BZG2**, and **BZG3**), we conducted an in-silico study of their physicochemical properties and toxicity risk (Table 4). The three compounds (**BZG1**, **BZG2**, and **BZG3**) do not present violations according to Lipinski’s^25^ and Veber’s^26^ rules (Table 4), following the requirements that indicate that these substances have drug-likeness features and have high bioavailability following oral administration.

**Table 4:** In silico prediction of physicochemical properties, absorption, distribution, excretion, and toxicity of the most promising compounds **BZG1**, **BZG2**, and **BZG3**.

Properties	Compounds		
	BZG1	BZG2	BZG3
Physicochemical			
MM (g/mol)	359.16	366.17	336.21
nHA	5	7	4
nHD	2	2	3
nRot	8	7	7
TPSA	62.72	96.63	58.07
LogS	− 3.99	− 5.18	− 0.90
LogP	3.13	3.56	2.47
Absorption			
Caco-2 permeability	No	No	Yes
P-gp Inhibitor	No	No	No
P-gp Substrate	No	Yes	Yes
HIA	No	Yes	Yes
Distribution			
VD	0.95 L/kg	0.51 L/kg	3.09 L/kg
BBB Penetration	No	Yes	Yes
Excretion			
T 1/2	Short	Short	Short
Toxicity			
hERG Blockers	Yes	No	No
H-HT	No	No	No
DILI	Yes	Yes	Yes
Carcinogenicity	No	No	No

MW: molecular weight; Log P: partition coefficient log; LogS: log of solubility; TPSA: topological polar surface area; nHA: hydrogen acceptors; nHD: hydrogen donor; nRot: rotatable bonds; HIA: gastrointestinal absorption; P-gp: P-glycoprotein; Caco-2: human colon carcinoma cell line; VD: distribution volume; BBB: blood–brain barrier; T1/2: half-life; hERG: human ether-a-go-go related gene; H-HT: Human Hepatotoxicity; DILI: Drug-Induced Liver Injury.

The parameters of gastrointestinal absorption, permeation of the Caco-2 cell line, and the blood–brain barrier can estimate the ability to permeate membranes. Except for **BZG1**, the other compounds are likely absorbed by the gastrointestinal tract. However, they may also enter the central nervous system, leading to adverse effects^27^. Scientists often use the Caco-2 cell line derived from human colon carcinoma, which has properties similar to intestinal epithelial cells, to predict the oral absorption of drugs^28^. Only the **BZG3** can permeate Caco-2 cell lines. In addition, the three compounds showed optimal volume distribution of 0.51–3.09 L/kg and short half-life (Table 4).

P-glycoprotein affects the pharmacokinetics of distinct drugs^29^, inhibition or induction of this protein lead to toxicity risks or may cause decrease in absorption at the intracellular level in cases of concomitant-administration therapies^30^. The in silico results indicate that these compounds are not P-gp inhibitors, but **BZG2** and **BZG3** can act as substrates (Table 4).

The toxicity profile of **BZG1**, **BZG2,** and **BZG3** was performed and indicated that these compounds have no carcinogenicity and human hepatotoxicity effects but showed the ability to induce liver injury. The hERG (human ether-a-go-go-related gene) measurement is usually used to predict proarrhythmic risk for new drugs^31^. In this case, only **BZG1** was demonstrated to act as hERG blockers, leading to an increased risk of fatal arrhythmias^32^ (Table 4).

Drug metabolism occurs on many sites, including enzymatically converting compounds through phase I reactions, such as oxidation, catalyzed by the Cytochrome P450 system^33^. We briefly investigated the metabolism of these substances against isoforms CYP1A2, CYP2C19, CYP2C9, CYP2D6, and CYP3A4, responsible for more than 90% of the oxidative metabolism of currently available drugs^34^. None of the compounds has been shown to act as a CYP inhibitor or substrate, thus not affecting oral bioavailability, plasma concentration, or increased toxicity and its half-life. Moreover, it is essential to mention that experimental data should be obtained to corroborate these predictions.

## Materials and methods

### Protein structure and LaSMMed chemical library

The study was performed using the crystallographic structures of two Mpro, one of which is in complex with an N3 ligand (PDB ID: 6LU7, chain A, resolution 2.16 Å)15 and the other with a ligand α-ketoamide (13b) (PDB ID: **6Y2F**, chain A, resolution 1.95 Å)^18^.

The missing residues of protein structures were added using the CHARMM-GUI platform (http://www.charmm-gui.org/)^35^. Subsequently, water molecules were removed, and the covalent bond between N3 and α-ketoamide ligands with the amino acid residue Cys145 was cleaved. After cleavage, the Cys145 amino acid residue and the inhibitors were reconstructed, making the necessary changes.

Next, the 3D structures from the LaSMMed Chemical Library were drawn using ChemDraw v.12.0 (PerkinElmer Informatics) and converted to pdbqt using Open Babel^36^. Finally, geometry optimization was performed using the MM2 force field implemented by ChemBio3D v.12.0 program (PerkinElmer Informatics).

### Molecular docking-based virtual screening

Virtual screening experiments were performed using AutoDock Vina 1.1.2^37^ and AutoDock4.2^38^. The protein structures were prepared in AutoDockTools (ADT) v.1.5.6^39^. The Laarckian Genetic Algorithm (LGA) was used to perform the simulation. We redocked peptide-like inhibitor N3 (6LU7) and alpha-ketoamide inhibitor O6K (6Y2F) to validate our protocol according to parameters previously described by our group^17^.

Both software returned ten binding poses per structure. The structures were selected through a consensus classification^40,41^. We consider 2 Å as an RMSD-based scoring, a metric for classifying structures based on poses obtained in virtual screening. The RMSD calculation was performed considering the pairs between the best poses of the structures obtained in the two crystallographic structures used in this study (6LU7 and 6Y2F). Mean RMSD was calculated to rank molecules according to the most conserved pose (i.e., less than 2 Å, better). The RMSD calculations were carried out using PyMOL software^42^.

### Molecular docking at allosteric sites in Mpro

The molecular docking in the allosteric sites followed the protocol used in the active site studies. We used AutoDock 4.2 and the crystallographic structure 6LU7. The allosteric site 1 (A1) comprises the residues Gln107, Pro108, Gly109, Ile200, Thr201, Val202, Asn203, Ile249, Pro252, Leu253, Phe294, Asp295 and Val297. Allosteric site 2 (A2) amino acids Phe3, Ile213, Asn214, Gln299, Cys300, Ser301, Tyr118, Phe140, and Leu141. Allosteric site 3 (A3) residues Leu271, Leu272, Gly275, Met276, and Asn277, as reported in the literature [49]. Each site was incorporated in the center of a grid box with a dimension of 60 × 60 × 60, and the spacing was 0.375 Å. The Laarckian Genetic Algorithm (LGA) was used to perform the simulation.

### Molecular dynamics simulations

Molecular dynamics (MD) simulations were carried out using GROMACS 2018.1 package^21^ with CHARMM36 force field^43^ from the best pose obtained by molecular docking. In addition, the enzyme topology preparation, calculation, and results analysis were carried out according to our group's description^17,44,45^. Each enzyme-ligand complex was inserted and centered in a periodic triclinic box (box dimensions: 3.982 × 4.337 × 5.086 nm; box volume: 702.68 nm^3^), solvated with water TIP3P type and then neutralized with sufficient positive (Na^+^) or negative (Cl^-^) ions.

### In vitro inhibition of rMpro proteolytic activity

Recombinant SARS-CoV-2 Mpro expressed in *E. coli* BL21(DE3)pLysS cells were used in a fluorescent resonance energy transfer (FRET) assay using the peptide DABCYL-AVLQ↓SGFRK-EDANS as substrate. The enzyme concentration was fixed at 1.5 μM, the substrate at 50 μM, and the compounds ranged from 1 to 10,000 μM. The enzyme and compounds were incubated in 5 mM NaCl and 20 mM Tris—HCl pH 8.0, 5 mM DTT for 15 min at 37 °C before starting with the substrate. The emission fluorescence of EDANS was monitored in the following parameters: λ_exc_ = 330 nm, λ_em_ = 490 nm, at 37 °C every 30 s for 45 min. Fluorescence data (RFU) was converted into substrate cleavage-specific activity using fluorescent conversion factor (FEC) previously calculated based on the EDANS-DABCYL fluorophore pair. Maximum enzyme activity was considered as the condition with vehicle (DMSO) in which the substrate cleavage rate was set as 100%, and the values were used to calculate the enzyme inhibition by the compounds. The concentration inhibiting 50% of the enzyme activity (IC_50_) and the standard deviation of the mean (SEM) were calculated using GraphPad Prism 9.0 v9.5.0 software.

### Lineaver-burk plot of Mpro inhibition BZG2

Mpro at 1.5 μM was incubated with vehicle or **BZG2** ranging from 10 to 150 μM (covering concentrations above and below IC_50_) for 15 min at 37 °C before starting with substrate addition at concentrations ranging from 50 to 1,056 μM. EDANS emission fluorescence was monitored at 37 °C, every 30 s for 45 min, with excitation and emission wavelengths set at λ_exc_ = 330 nm and λ_em_ = 490 nm, respectively. RFU data was converted into cleaved substrate concentration using FEC. After conversion, the inverse of the enzyme's maximum velocity immediately preceding the saturation point was plotted against the inverse of substrate concentration, followed by linear regression of each condition tested to construct a Lineweaver–Burk plot. The calculations were performed using GraphPad Prism 9.0 v9.5.0 software.

### In silico predictions of pharmacokinetic and toxicity parameters (ADME-Tox)

Predictions of the compounds BZG1, BZG2, and BZG3's in silico drug-likeness, pharmacokinetic, and toxicity parameters were assessed by rule-based filters from Lipinski^25^ and Veber^26^ using the ADMETLab 2.0 platform (https://admetmesh.scbdd.com/)^46^.

## Conclusion

Our research has discovered three benzoylguanidines that exhibit binding affinities with Mpro. Our molecular dynamics simulations have demonstrated that **BZG1** remains stable, while **BZG3** moves towards the active site of Mpro. **BZG2** has shown selectivity towards allosteric sites and has been confirmed to exhibit uncompetitive type inhibition through in vitro tests. Our in silico drug-likeness predictions, pharmacokinetic, and toxicity parameters have indicated that all three compounds have favorable properties and can potentially be used as antiviral drugs. These promising results highlight the potential of benzoylguanidines as effective inhibitors of SARS-CoV-2 Mpro, providing valuable insights into their drug-like properties.

### Supplementary Information

Supplementary Information.

## Data Availability

The authors can confirm that all relevant data are included in the article.
