# Supplementary material for: Molecular modelling studies and in vitro enzymatic assays identified A 4-(nitrobenzyl)guanidine derivative as inhibitor of SARS-CoV-2 Mpro
Source: Sci Rep. 2024 Apr 14;14:8620. doi: 10.1038/s41598-024-59292-0 (PMC11016540; doi:10.1038/s41598-024-59292-0)
Supplement: Supplementary file 1 — Supplementary Information. [file 41598_2024_59292_MOESM1_ESM.docx]

**Molecular Modelling Studies And In Vitro Enzymatic Assays Identified A 4-(Nitrobenzyl)Guanidine Derivative As Inhibitor of SARS-CoV-2 Mpro**

Kaio Maciel de Santiago-Silva^a^, Priscila Goes Camargo^a^, Larissa Esteves Carvalho Constant^c^, Stephany da Silva Costa^c^, Giovanna Barbosa Frensel^c^, Diego Allonso Rodrigues dos Santos da Silva^c^, Gerson Nakazato^d^, Camilo Henrique da Silva Lima^b^, Marcelle de Lima Ferreira Bispo^a^*.

^a^Laboratório de Síntese de Moléculas Medicinais (LaSMMed), Departamento de Química, Centro de Ciências Exatas, Universidade Estadual de Londrina, Londrina, Brazil.

^b^Departamento de Química Orgânica, Instituto de Química, Universidade Federal do Rio de Janeiro, Rio de Janeiro, Brazil

^c^Departamento de Biotecnologia Farmacêutica, Faculdade de Farmácia, Universidade Federal do Rio de Janeiro, Rio de Janeiro, RJ 21941-902, Brazil

^d^Departamento de Microbiologia, Centro de Ciências Biológicas, Universidade Estadual de Londrina, Londrina, Brazil;

**SUMMARY**

**Pg 1. Table S1**. SMILES of the 86 structures used in the virtual screening.

**Pg 3. Table S2**. Top 20 ranked **BZG** structures selected by virtual screening on 6LU7 and 6Y2F Mpro enzyme.

**Pg. 3.** **Table S3**. RMSD of **BZG** binding poses selected by virtual screening on 6LU7 and 6Y2F Mpro enzyme.

**Pg. 4.** **Figure S1.** Cα-RMSD analysis of Mpro in the apo form S1+S1’ (red), S2 (green), S4 (blue), S5 (orange), A1 (purple), A2 (yellow) and A3 (pink).

**Table S1**. SMILES of the 86 structures used in the virtual screening.

| **BZG** | **SMILES** |
| --- | --- |
| 1 | O=C(N/C(NC1=CC=C(OC)C=C1)=N\CC2=CC=CC=C2)C3=CC=CC=C3 |
| 2 | O=C(N/C(NC1=CC=C([N+]([O-])=O)C=C1)=N/C2CCCCC2)C3=CC=CC=C3 |
| 3 | O=C(N/C(NCC1=CC=CC=C1)=[NH+]/C2CCCCC2)C3=CC=CC=C3 |
| 4 | O=C(/N=C(NC1=CC=C(OC)C=C1)/NC2=CC=CC=C2)C3=CC=CC=C3 |
| 5 | O=C(NC(NC1CCCCC1)=NC2=CC=CC=C2)C3=CC=CC=C3 |
| 6 | O=C(/[NH+]=C(NC1=CC=CC=C1)/NC2CCCCC2)C3=CC=CC=C3 |
| 7 | O=C(N/C(NC1=CC=CC=C1)=N/C2CCCCC2)C3=CC=CC=C3 |
| 8 | O=C(/[NH+]=C(NCC1=CC=CC=C1)/NCC2=CC=CC=C2)C3=CC=CC=C3 |
| 9 | O=C(N/C(NCC1=CC=CC=C1)=[NH+]/CC2=CC=CC=C2)C3=CC=CC=C3 |
| 10 | O=C(N/C(NCC1=CC=CC=C1)=[NH+]/CC2=CC=CC=C2)C3=CC=CC=C3 |
| 11 | O=C(/N=C(NC1=CC=CC=C1)/NCC2=CC=CC=C2)C3=CC=CC=C3 |
| 12 | O=C(N/C(NC1=CC=CC=C1)=N\CC2=CC=CC=C2)C3=CC=CC=C3 |
| 13 | O=C(N/C(NCC1=CC=CC=C1)=N/C2=CC=CC=C2)C3=CC=CC=C3 |
| 14 | O=C(NC(NC1=CC=CC=C1)=NC2=CC=CC=C2)C3=CC=CC=C3 |
| 15 | O=C(/N=C(NC1=CC=CC=C1)\NC2=CC=CC=C2)C3=CC=CC=C3 |
| 16 | O=C(N/C(NC1=CC=CC=C1)=N\C2=CC=CC=C2)C3=CC=CC=C3 |
| 17 | O=C(/[NH+]=C(NC1=CC=C(OC)C=C1)/NC2CCCCC2)C3=CC=CC=C3 |
| 18 | O=C(N/C(NC1CCCCC1)=N\C2=CC=C(OC)C=C2)C3=CC=CC=C3 |
| 19 | O=C(N/C(NC1=CC=C(OC)C=C1)=N/C2CCCCC2)C3=CC=CC=C3 |
| 20 | O=C(/N=C(NC1=CC=C(OC)C=C1)/NCC2=CC=CC=C2)C3=CC=CC=C3 |
| 21 | O=C(N/C(NCC1=CC=CC=C1)=N/C2=CC=C(OC)C=C2)C3=CC=CC=C3 |
| 22 | O=C(/N=C(NC1=CC=C([N+]([O-])=O)C=C1)/NCC2=CC=CC=C2)C3=CC=CC=C3 |
| 23 | O=C(N/C(NCC1=CC=CC=C1)=N/C2=CC=C([N+]([O-])=O)C=C2)C3=CC=CC=C3 |
| 24 | O=C(N/C(NC1=CC=C([N+]([O-])=O)C=C1)=N/CC2=CC=CC=C2)C3=CC=CC=C3 |
| 25 | O=C(/N=C(NC1=CC=C([N+]([O-])=O)C=C1)/NC2CCCCC2)C3=CC=CC=C3 |
| 26 | O=C(N/C(NC1CCCCC1)=N/C2=CC=C([N+]([O-])=O)C=C2)C3=CC=CC=C3 |
| 27 | O=C(N/C(NC1=CC=C([N+]([O-])=O)C=C1)=N\C2CCCCC2)C3=CC=CC=C3 |
| 28 | O=C(NC(NC1=CC=C([N+]([O-])=O)C=C1)=NC2=CC=CC=C2)C3=CC=CC=C3 |
| 29 | O=C(/N=C(NC1=CC=CC=C1)/NC2=CC=C([N+]([O-])=O)C=C2)C3=CC=CC=C3 |
| 30 | O=C(N/C(NC1=CC=CC=C1)=N/C2=CC=C([N+]([O-])=O)C=C2)C3=CC=CC=C3 |
| 31 | O=C(NC(N)=[NH2+])C1=CC=CC=C1 |
| 32 | O=C(/[NH+]=C(N)/N)C1=CC=CC=C1 |
| 33 | O=C(NC(NCCC1=CC=CC=C1)=[NH2+])C2=CC=CC=C2 |
| 34 | O=C(/[NH+]=C(N)/NCCC1=CC=CC=C1)C2=CC=CC=C2 |
| 35 | O=C(N/C(N)=N/CCC1=CC=CC=C1)C2=CC=CC=C2 |
| 36 | O=C(NC(NC1=CC=CC=C1)=N)C2=CC=CC=C2 |
| 37 | O=C(/N=C(N)/NC1=CC=CC=C1)C2=CC=CC=C2 |
| 38 | O=C(N/C(N)=N/C1=CC=CC=C1)C2=CC=CC=C2 |
| 39 | O=C(NC(NC1=C(OC)C=CC=C1)=N)C2=CC=CC=C2 |
| 40 | O=C(/N=C(N)/NC1=C(OC)C=CC=C1)C2=CC=CC=C2 |
| 41 | O=C(N/C(N)=N/C1=C(OC)C=CC=C1)C2=CC=CC=C2 |
| 42 | O=C(NC(NC1=CC(OC)=CC=C1)=N)C2=CC=CC=C2 |
| 43 | O=C(/N=C(N)/NC1=CC(OC)=CC=C1)C2=CC=CC=C2 |
| 44 | O=C(N/C(N)=N/C1=CC(OC)=CC=C1)C2=CC=CC=C2 |
| 45 | O=C(/N=C(NC1=CC=C(OC)C=C1)/N)C2=CC=CC=C2 |
| 46 | O=C(NC(NC1=CC=C(OC)C=C1)=N)C2=CC=CC=C2 |
| 47 | O=C(N/C(N)=N/C1=CC=C(OC)C=C1)C2=CC=CC=C2 |
| 48 | O=C(/N=C(NC1=CC=C([N+]([O-])=O)C=C1)/NC2CCCCC2)C3=CC=CC=C3 |
| 49 | O=C(NC(NC1CCCCC1)=NC2=CC=C([N+]([O-])=O)C=C2)C3=CC=CC=C3 |
| 50 | O=C(/N=C(NC1=CC=C([N+]([O-])=O)C=C1)/NC2=CC=CC=C2)C3=CC=CC=C3 |
| 51 | O=C(NC(NC1=CC=CC=C1)=NC2=CC=C([N+]([O-])=O)C=C2)C3=CC=CC=C3 |
| 52 | O=C(N/C(NC1=CC=C([N+]([O-])=O)C=C1)=N/C2=CC=CC=C2)C3=CC=CC=C3 |
| 53 | O=C(/[NH+]=C(NC1CCCCC1)/NCC2=CC=CC=C2)C3=CC=CC=C3 |
| 54 | O=C(N/C(NC1CCCCC1)=[NH+]\CC2=CC=CC=C2)C3=CC=CC=C3 |
| 55 | O=C(/N=C(NC1=CC=C(OC)C=C1)/NCC)C2=CC=CC=C2 |
| 56 | O=C(N/C(NCC)=N/C1=CC=C(OC)C=C1)C2=CC=CC=C2 |
| 57 | O=C(N/C(NC1=CC=C(OC)C=C1)=N/CC)C2=CC=CC=C2 |
| 58 | O=C(/[NH+]=C(NCC1=CC=C(OC)C=C1)/NCC2=CC=CC=C2)C3=CC=CC=C3 |
| 59 | O=C(N/C(NCC1=CC=CC=C1)=[NH+]/CC2=CC=C(OC)C=C2)C3=CC=CC=C3 |
| 60 | O=C(N/C(NCC1=CC=C(OC)C=C1)=[NH+]/CC2=CC=CC=C2)C3=CC=CC=C3 |
| 61 | O=C(/[NH+]=C(N)/NCC1=CC=CC=C1)C2=CC=CC=C2 |
| 62 | O=C(NC(NCC1=CC=CC=C1)=[NH2+])C2=CC=CC=C2 |
| 63 | O=C(N/C(N)=[NH+]/CC1=CC=CC=C1)C2=CC=CC=C2 |
| 64 | O=C(/N=C(NC1=CC=C(O)C=C1)/N)C2=CC=CC=C2 |
| 65 | O=C(NC(NC1=CC=C(O)C=C1)=N)C2=CC=CC=C2 |
| 66 | O=C(N/C(N)=N/C1=CC=C(O)C=C1)C2=CC=CC=C2 |
| 67 | O=C(/N=C(NC1=CC=C(Cl)C=C1)/N)C2=CC=CC=C2 |
| 68 | O=C(NC(NC1=CC=C(Cl)C=C1)=N)C2=CC=CC=C2 |
| 69 | O=C(N/C(N)=N/C1=CC=C(Cl)C=C1)C2=CC=CC=C2 |
| 70 | O=C(/[NH+]=C(NCC1=CC=CC=C1)/N)C2=CC=CC=C2 |
| 71 | O=C(/N=C(NC1=CC=C([N+]([O-])=O)C=C1)/N)C2=CC=CC=C2 |
| 72 | O=C(NC(NC1=CC=C([N+]([O-])=O)C=C1)=N)C2=CC=CC=C2 |
| 73 | O=C(N/C(N)=N/C1=CC=C([N+]([O-])=O)C=C1)C2=CC=CC=C2 |
| 74 | O=C(/[NH+]=C(NCCCC)/N)C1=CC=CC=C1 |
| 75 | O=C(NC(NCCCC)=[NH2+])C1=CC=CC=C1 |
| 76 | O=C(N/C(N)=[NH+]/CCCC)C1=CC=CC=C1 |
| 77 | O=C(/[NH+]=C(NC1CCCCC1)/NC2=CC=CC=C2)C3=CC=CC=C3 |
| 78 | O=C(/[NH+]=C(NCCCCCC)/N)C1=CC=CC=C1 |
| 79 | O=C(NC(NCCCCCC)=[NH2+])C1=CC=CC=C1 |
| 80 | O=C(N/C(N)=[NH+]/CCCCCC)C1=CC=CC=C1 |
| 81 | O=C(/[NH+]=C(NCC)/N)C1=CC=CC=C1 |
| 82 | O=C(NC(NCC)=[NH2+])C1=CC=CC=C1 |
| 83 | O=C(N/C(N)=[NH+]/CC)C1=CC=CC=C1 |
| 84 | O=C(/[NH+]=C(NC)/N)C1=CC=CC=C1 |
| 85 | O=C(NC(NC)=[NH2+])C1=CC=CC=C1 |
| 86 | O=C(N/C(N)=[NH+]/C)C1=CC=CC=C1 |
| 87 | O=C(/[NH+]=C(N1CCCCC1)\NC2=CC=CC=C2)C3=CC=CC=C3 |
| 88 | O=C(NC(N1CCCCC1)=NC2=CC=CC=C2)C3=CC=CC=C3 |

**Table S2**. Top 20 ranked **BZG** structures selected by virtual screening on 6LU7 and 6Y2F Mpro enzyme.

| **6LU7** | |  | **6Y2F** | |
| --- | --- | --- | --- | --- |
| **BZG** | **Energy (kcal/mol)** |  | **BZG** | **Energy (kcal/mol)** |
| **12** | -8,9 |  | **28** | -7,9 |
| **75** | -8,8 |  | **29** | -7,8 |
| **28** | -8,6 |  | **6** | -7,7 |
| **29** | -8,5 |  | **17** | -7,6 |
| **6** | -8,4 |  | **30** | -7,6 |
| **17** | -8,3 |  | **24** | -7,5 |
| **50** | -8,12 |  | **50** | -7,4 |
| **25** | -8,11 |  | **1** | -7,3 |
| **9** | -8,1 |  | **23** | -7,3 |
| **13** | -8,1 |  | **27** | -7,3 |
| **1** | -8,1 |  | **41** | -7,3 |
| **46** | -8,1 |  | **3** | -7,3 |
| **26** | -8 |  | **50** | -7,3 |
| **3** | -8 |  | **2** | -7,15 |
| **11** | -7,9 |  | **25** | -7,14 |
| **48** | -7,9 |  | **12** | -7,12 |
| **57** | -7,9 |  | **75** | -7,11 |
| **5** | -7,8 |  |  |  |
| **16** | -7,8 |  |  |  |
| **2** | -7,8 |  |  |  |

The blue color indicated the consensus **BZG** between the two Mpro structures.

**Table S3**. RMSD of **BZG** binding poses selected by virtual screening on 6LU7 and 6Y2F Mpro enzyme.

| **BZD** | **RMSD (Å)** |
| --- | --- |
| 3 | 0.588 |
| 1 | 0.764 |
| 2 | 0.936 |
| 17 | 2.458 |
| 28 | 5.014 |
| 12 | 5.180 |
| 6 | 5.288 |
| 75 | 5.432 |
| 29 | 5.909 |
| 25 | 6.041 |
| 50 | 6.331 |


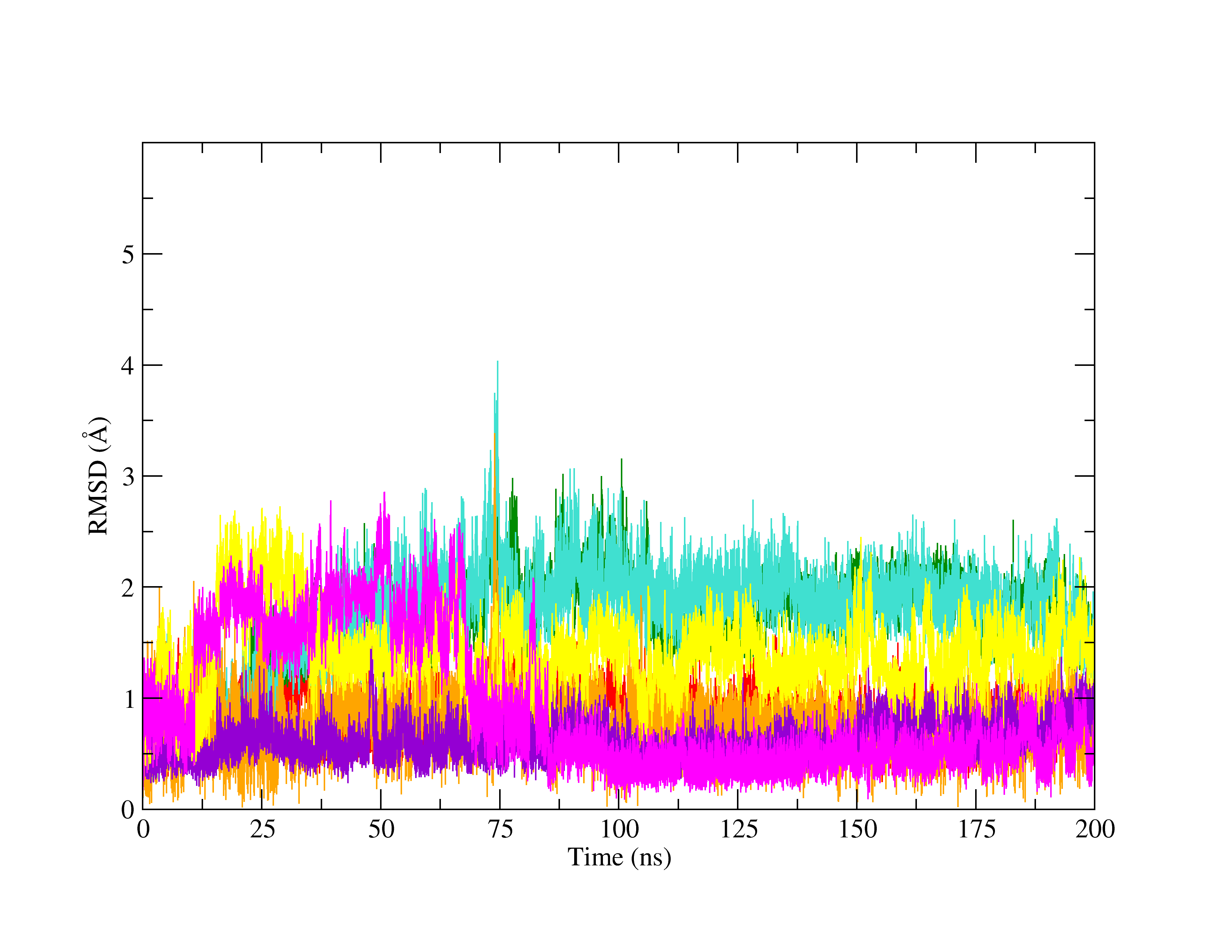


**Figure S1.** Cα-RMSD analysis of Mpro in the apo form S1+S1’ (red), S2 (green), S4 (blue), S5 (orange), A1 (purple), A2 (yellow) and A3 (pink).
